# Prophylactic cranial irradiation in locally advanced non-small cell lung cancer: outcome of recursive partitioning analysis group 1 patients

**DOI:** 10.1186/1756-9966-27-80

**Published:** 2008-12-04

**Authors:** Ali Aydin Yavuz, Erkan Topkan, Cem Onal, Melek Nur Yavuz

**Affiliations:** 1 Baskent University Medical Faculty, Adana Medical and Research Center, Department of Radiation Oncology, Kisla Saglik Yerleskesi, Adana, Turkey

## Abstract

**Background:**

Prophylactic cranial irradiation (PCI) has been demonstrated to reduce or delay the incidence of brain metastases (BM) in locally advanced non-small cell lung carcinoma (LA-NSCLC) patients with various prognostic groups. With this current cohort we planned to evaluate the potential usefulness of prophylactic cranial irradiation (PCI) specifically in recursive partitioning analysis (RPA) Group 1, which is the most favorable group of LA-NSCLC patients.

**Methods:**

Between March 2007 and February 2008, 62 patients in RPA group 1 were treated with sequential chemoradiotherapy and PCI for stage IIIB NSCLC. The induction chemotherapy consisted of 3 courses of cisplatin (80 mg/m^2^) and docetaxel (80 mg/m^2^); each course was given every 21 days. Thoracic radiotherapy (TRT) was given at a dose of 60 Gy using 3-D conformal planning. All patients received a total dose of 30 Gy PCI (2 Gy/fr, 5 days a week), beginning on the first day of the TRT. Then, all patients received 3 further courses of the same chemotherapy protocol.

**Results:**

Six (9.7%) patients developed brain metastases during their clinical course. Only one (2%) patient developed brain metastasis as the site of first treatment failure. Median brain metastasis-free survival, overall survival, and progression free survival were 16.6, 16.7, and 13.0 months, respectively. By univariate analysis, rates of BM were significantly higher in patients younger than 60 years of age (p = 0.03). Multivariate analysis showed no significant difference in BM-free survival according to gender, age, histology, and initial T- and N-stage.

**Conclusion:**

The current finding of almost equal bone metastasis free survival and overall survival in patients with LA-NSCLC in RPA group 1 suggests a longer survival for patients who receive PCI, and thereby have a reduced risk of BM.

## Background

Brain metastases (BM) are a common complication of locally advanced non-small cell lung cancer (LA-NSCLC), especially in patients who undergo radical treatment protocols. Of these patients, 21% to 54% develop BM during the course of their disease. [1-3]. and another 15–30% carry a risk of the first treatment failure occurring in the brain. [4-7]. Studies have shown that the addition of chemotherapy to radiation therapy (RT) reduces extracranial distant metastases. [5]. and improves survival. [8,9]. but does not alter brain relapse rates. [5]. which emphasizes the need for treatment directed at BM micrometastases. In selected non-randomized [1,4,10-12]. and randomized studies. [6,7,13,14]. prophylactic cranial irradiation (PCI) has been demonstrated to reduce the incidence or delay the onset of BM in patients with LA-NSCLC after primary therapy.

Several factors including histology, stage, duration of survival, performance status, chemotherapy protocol, age at presentation, and sex have been associated with risk of BM development. [2,13,15-18]. In earlier studies, recursive partitioning analysis (RPA) classification was demonstrated to be a useful tool for predicting survival in patients with LA-NSCLC. [19,20]. Survival analysis revealed that RPA classification identified five distinct subgroups with significantly different median survival times, ranging from 2.9 mo in Group 5 to 16.2 mo in Group 1. [20] (Table 1). In addition, longer survival of patients with LA-NSCLC treated with radiation alone or radiation plus chemotherapy was associated with an increased incidence of CNS metastases, according to a review of data from the Radiation Therapy Oncology Group (RTOG) studies. [1,2,19]. Based on these different analyses, it is reasonable to assume that many patients with LA-NSCLC do not live long enough to develop brain failure. We hypothesized that studies including patients with various RPA groups may not reflect the true value of PCI in specific groups; thus, with the current cohort we specifically evaluated the role of PCI in RPA Group 1 (Karnofsky performance status ≥ 90 and previously treated by chemotherapy).

**Table 1:** Radiation Therapy Oncology Group (RTOG) recursive partitioning analysis (RPA) groups in patients with locally-advanced non-small-cell lung cancer (LA-NSCLC).

**RPA Group**	**Definition**	**Median survival (months)**
I	KPS of > or = 90, who received chemotherapy	16.2
II	KPS of > or = 90, who received no CT, but had no PE	11.9
III	KPS < 90, younger than 70 years, with non-large cell histology	9.6
IV	KPS > or = 90, but with PE, or KPS < 90, younger than 70 years, and with large cell histology, or older than 70 years, but without PE	5.6–6.4
V	older than 70, with PE	2.9

Abbreviations: KPS: Karnofsky Performance Status; CT: Chemotherapy; PE: Pleural effusion.

## Methods

### Patients

This retrospective analysis included 62 patients with a histological diagnosis of LA-NSCLC (stage IIIB) meeting the following criteria; age older than 18 and younger than 70 years, RPA Group 1 (Karnofsky Performance Status (KPS) ≥ 90, previously treated by cisplatin-based chemotherapy), no superior sulcus tumor, no progressive disease following induction chemotherapy, no prior history of thoracic and cranial RT, no more than 10% weight loss in the last 6 months, and signed written informed consent, those treated at our institution between March 2007 and February 2008. Further staging procedures included laboratory investigations, computed tomography of the thorax and abdomen, bone scintigraphy, pulmonary function tests, and baseline magnetic resonance imaging of the brain showing no suspicion for intracranial metastases. This study was formally approved by the Baskent University's institutional review board before collection of all patient information. Pretreatment patient and tumor characteristics are shown in Table 2.

**Table 2:** Patient and tumor characteristics.

**Characteristic**	**n (%)**
Gender	
Male	48 (77.4)
Female	14 (22.6)
Age (y)	
Median (range)	59.6 (38–69)
< 60	26 (41.9)
≥ 60	36 (58.1)
Histology	
Epidermoid	51 (82.3)
Adenocarcinoma	11 (17.7)
T-stage	
1	0 (0)
2	13 (21.0)
3	12 (19.3)
4	37 (59.7)
N-stage	
0	4 (6.5)
1	13 (21.0)
2	19 (30.6)
3	26 (41.9)
TN-stage	
T4, N0	4 (6.5)
T4, N1	13 (21.0)
T4, N2	18 (29.0)
T4, N3	3 (4.8)
T3, N3	11 (17.7)
T2, N3	13 (21.0)
Response to induction CT	
CR	11 (17.7)
PR	28 (45.2)
SD	23 (37.1)

Abbreviations: T: Tumor; N: Node; CT: Chemotherapy; CR: Complete response; PR: Partially response; SD: Stable disease.

### Chest Irradiation

Three-dimensional conformal radiation therapy (3D-CRT) was used in all patients. The treatment planning for eligible patients was based on gross tumor volume (GTV), which was restricted to all primary tumors and abnormally enlarged hilar or mediastinal lymph nodes greater than 1 cm in diameter seen on CT images or metabolically active areas on PET-CT. Clinical target volumes (CTVs) were defined by adding 1-cm margins to GTVs. Planning target volume-1 (PTV1) was created by adding an addition 1.5 cm margin to CTVs, and PTV2 (boost field) was defined as the GTVs plus a 1.5-cm margin. Three-dimensional CRT was performed to minimize the volume of normal lung and surrounding normal tissues irradiated while providing coverage of PTVs by at least 95% isodose surfaces; the "100% isodose" (prescription doses) were defined at each isocenter. Thoracic irradiation was given through the anteroposterior-posteroanterior (AP-PA) portals with individualized multileaf collimator blocks for PTV1 up to 46 Gy, followed by off-spinal cord oblique portals up to 60 Gy (14 Gy boost) for PTV2, in a sequential manner. All patients received daily treatment five days a week, using 2 Gy fractions administered using linear accelerators with 6 MV or 18 MV photon energies (Clinac DBX-1031, and/or DHX-3323, Varian Medical Systems, Palo Alto, CA, USA). On the initial day of treatment and every week throughout the treatment, course field location was confirmed by comparing the digitally reconstructed radiographs and the portal images.

### Prophylactic cranial irradiation

Patients were simulated in a supine position with their heads fixed with thermoplastic head masks; radio-opaque markers were placed at the lateral orbital canthi to assist in blocking the lenses. The target volume consisted of all the intracranial contents with at least a 1-cm margin around the bony skull at each margin. The inferior border at the cervical vertebral bodies was the C1-C2 interspace. Individually shaped radiation fields with multileaf collimators were used to define the irradiation target volume and exclude tissues that were not to be irradiated. Patients were treated on a megavoltage linear accelerator with 6 MV photons. All patients received PCI at 2 Gy per fraction, 5 days per week, for 3 weeks, with a total dose of 30 Gy, beginning on the first day of the thoracic irradiation. Treatment was delivered with equally weighted right and left lateral fields with the dose calculated on the central ray at mid-separation of the beams.

### Chemotherapy

All 62 patients received 3 courses of induction chemotherapy involving cisplatin (80 mg/m^2^) and docetaxel (80 mg/m^2^) every 21 days, prior to RT. After completion of RT, all patients received 3 further courses of the same chemotherapy regimen. Chemotherapy was not permitted during the course of RT.

### Patient evaluation

Patients were examined by a radiation oncologist once a week during the RT course. After completion of RT, patients were followed at bimonthly intervals or more frequently if necessary. Besides the standard work-up for locoregional and distant metastases, an MRI of the brain was performed at bimonthly intervals or more frequently if there was a suspicion of BM during the follow-up period.

### Statistics

Variables analyzed were age at the time of diagnosis, gender, histological type (squamous cell carcinoma versus adenocarcinoma), and T-status (T1-2 versus 3–4), N-status (N0-1 versus 2–3). Univariate analyses were performed first for each variable to compare the BM+ and BM- groups using a Chi-square test. The primary endpoint was BM-free survival (BMFS), calculated as the time between the first day of the PCI and the date of BM diagnosis. Similarly, overall survival (OS) was calculated as the time between the first day of the PCI and the date of death or last visit. Estimates of 1-year and 18-month survival were calculated by the Kaplan-Meier method, with comparisons between subsets performed with two-sided log-rank tests. All tests were two-tailed, with a p-value < 0.05 considered significant.

## Results

**Figure 1:**
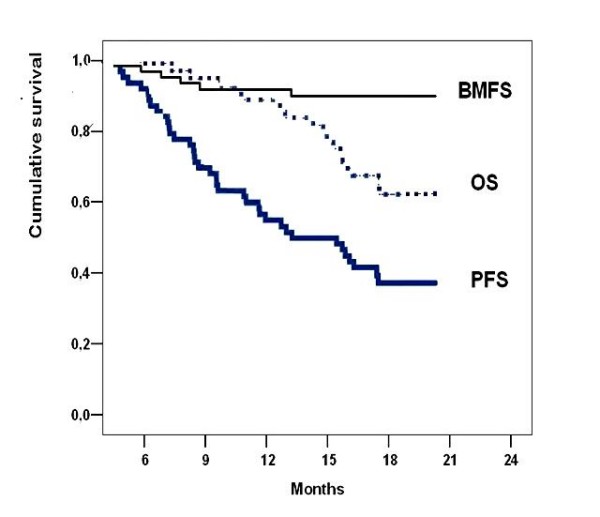
**Survival data of patients with locally advanced non-small cell lung cancer in recursive partitioning analysis group 1 treated with PCI.** BMFS = Brain-metastasis free survival, OS = Overall survival, PFS = Progression free survival.

At a median follow-up of 16.8 months (range: 7–18.6 months), 41 of the 62 patients with LA-NSCLC were alive at the time of analysis. Median survival for the entire population was 16.7 months (SE: 0.51; 95% CI: 15.7–17.7 months). As illustrated in Figure 1, the 1-year, and 18-month estimates of the OS were 86% and 62%, respectively. Six patients (9.7%) developed brain metastases at some time during the course of follow-up. Only one (2%) patient developed brain metastasis as the site of first failure. The median time to the development of brain metastases was 7 months (range, 5–13 months). Brain metastasis was the only site of recurrence in one of six patients. Among the other five patients with brain metastasis, local progression, liver metastasis, and adrenal metastasis developed asynchronously in four, two, and one patient, respectively. Median BMFS was 16.7 months (SE:0.51, 95% CI: 15.5–17.7 months). Two of the six patients with brain metastasis were alive at the time of analysis. As illustrated in Figure 1, BMFS rates at 1 year and 18 months were 92% and 90%, respectively.

Sites of extracranial failure included liver in eight (12.9%) patients, bone in five (8.1%), lung in four (6.5%), adrenal in three (4.8%), and more than one site in two (3.2%). A total of 23 (37.1%) patients developed locoregional tumor progression during follow-up. Median progression-free survival was 13.0 months (SE: 2.1; 95% CI: 8.9–17.0) months. The estimates of 1-year and 18-month progression-free survival rates for the entire patient population were 54% and 37%.

By univariate analysis, brain metastasis was significantly higher in patients with age < 60 years (p = 0.03). An exploratory analysis of prognostic parameters using a Cox proportional hazards model showed no significant difference in BMFS according to gender, age, histology, and initial T- and N-stage (p > 0.05 for all; Table 3).

**Table 3:** Multivariate analyses of predictive factors for brain metastases following prophylactic cranial irradiation in patients with locally advanced non-small cell lung cancer in recursive partitioning analysis group 1.

Variable	Estimate (mo)	SE	95% CI	p-value
Gender (female)	15.7	0.56	14.6–16.8	0.28
Age (< 60)	15.0	0.62	13.8–16.2	0.15
Histology (epidermoid)	16.6	0.58	15.4–17.7	0.16
T-stage (3–4)	16.9	0.63	15.6–18.4	0.92
N-stage (2–3)	16.2	0.65	15.0–17.5	0.07

Abbreviations: T: Tumor; N: Node

## Discussion

Several recent studies have reported excellent median and two-year survival rates of 15–25 months and 37–66%, respectively, in patients with LA-NSCLC who receive multi-modal therapy. [1-5,19-25]. Incorporation of systemic chemotherapy was associated with a significant reduction in the incidence of extracranial metastases and longer survival; however this was also associated with increased rates of overall brain failure (21–54%) and brain as the first site of relapse (15–30%). [1-7,19,25]. These results emphasize the significance of treatment failure localized to the brain in patients with prolonged survival after aggressive treatment for LA-NSCLC. This finding has prompted the inclusion of PCI in some clinical studies. [1,4,10].

PCI, as administered in our protocol, demonstrated efficacy in reducing the incidence of overall BM and the brain as the first site of relapse (9.7% and 2%, respectively). This is in accordance with the results of earlier randomized and non-randomized studies of combined therapies [1,4,6,7,10-14]. summarized in Table 4. To our knowledge, the current study is the first attempt to specifically address the role of PCI in patients with LA-NSCLC in RPA Group 1. Despite the non-randomized nature and relatively short follow-up interval of our study, the BMFS and OS were almost equal, which suggests a longer survival for patients with LA-NSCLC who have a reduced BM risk from PCI protocols such as the one used here. In general, except for the study published by Pöttgen et al. [14]. in which only patients with stage IIIA NSCLC were studied, most studies included patients with disease in various stages and different histology, including adenosquamous and large cell tumors. In contrast, the current cohort included only patients with unresectable stage IIIB disease with epidermoid or adenocarcinoma histology.

**Table 4:** Studies of prophylactic cranial irradiation for patients with locally advanced non-small cell lung cancer in recursive partitioning analysis group 1.

Reference	N patients	Histology	Primary Tx	Dose of PCI	BM PCI- (%)	BM PCI+ (%)	P-value
Albain et al.^1^	126	All NSCLC	Trimodality	36 Gy (2 Gy/fr)	16	8	0.36
Strauss et al.^4^	54	Non-squamous	Trimodality	30 Gy (2 Gy/fr)	12	0	0.32
Skarin et al.^12^	41	All NSCLC	Trimodality	NS	27	14	
Rusch et al.^11^	75	NS	Ctx+TRT	36 Gy (2 Gy/fr) 30 Gy (3 Gy/fr)	-	0	-
Stuschke et al.^10^	75	All NSCLC	Trimodality	30 Gy (2 Gy/fr)	54	13	0.0004
Cox et al.^5^	281	All NSCLC	TRT	20 Gy (2 Gy/fr)	13	6	0.038
Unsawasdi.^7^	97	All NSCLC	Trimodality Ctx+TRT	30 Gy (3 Gy/fr)	27	4	0.002
Russell et al.^6^	187	Non-squamous	TRT	30 Gy (3 Gy/fr)	19	9	0.06
Mira et al.^32^	111	All NSCLC	Ctx + TRT	30 Gy (2 Gy/fr) 37.5 Gy (2.5Gy/fr)	11	0	0.0001
Pöttgen et al.^14^	112	All NSCLC (Stage IIIA)	Trimodality	30 Gy (2 Gy/fr)	27.2	9.1	0.02 0.04
Yavuz et al.*	62	Squamous and adeno ca (Stage IIIB)	Ctx + TRT	30 Gy (2 Gy/fr)	-	9.7	-

(*) Current study.

Abbreviations: Tx = treatment; NSCLC = non-small cell lung cancer, Ctx = chemotherapy, TRT = thoracic radiotherapy; PCI = prophylactic cranial irradiation, BM = brain metastasis.

A recent retrospective review of the Southwest Oncology Group (SWOG) database reported by Gaspar et al.[23]. included 422 patients with stage IIIA/IIIB: 268 (64%) experienced disease progression; 54 (20%) had progression in the brain only and 17 (6.5%) in the brain and other sites simultaneously. Time from treatment to disease progression in the brain in 71 patients was as follows: during treatment, 16 relapses (22.5%); 0 to 16 weeks after treatment, 17 relapses (24%); 16 weeks to 6 months after treatment, 10 relapses (14%); 6 to 12 months after treatment, 16 relapses (22.5%), and more than 12 months after treatment, 12 relapses (17%). Thus, 83% of all brain relapses manifest in the first 12 months after treatment. Similarly, the median time to the development of brain metastases was 7 months (range, 5–13 months) in our study. Based on these results, although longer follow up is needed, we reasonably estimate that most of the brain relapses in our current cohort presented during the follow-up period, and potentially only a limited number of patients will subsequently be further affected; also, the majority of additional deaths will be associated with local progression and/or extracranial metastases, rather than brain failures.

In previously reported PCI studies, the tested total radiation dose and dose per fraction have been tested in previous studies and ranged from 20 Gy to 36 Gy and 2 Gy to 4 Gy, respectively. [1,4,6,7,10-14]. However, radiation regimens for PCI that have positively influenced patterns of CNS failures have included total doses of 30–36 Gy and fractional doses of 2–3 Gy. [1,4,6,10,14]. For the current study, we chose a smaller fractional dose of 2 Gy and a total dose of 30 Gy to minimize late tissue toxicity, as preferred in PCI protocols in small cell lung cancer (SCLC). In a previous study, Stuschke et al. [10]. demonstrated that this regimen effectively reduced BM from 54% to 13%, with no differences in neuropsychologic testing in PCI versus non-PCI patients at 4 years. Furthermore, this 75% reduction in the rate of BM is thought to lead to an 8% improvement in OS. [31]. Enlightened with the present evidence, it is reasonable to expect longer survival in patients with radically treated LA-NSCLC who recieve PCI. However, to achieve more firm conclusions, these estimates regarding the toxicity of PCI and its potential impact on survival need to be confirmed with larger randomized studies. One such study is the RTOG 0214 trial, which is a large-scale randomized phase III trial comparing PCI (2 Gy/fraction, totally 30 Gy in three weeks) versus observation in patients with LA-NSCLC following the completion of definitive locoregional/systemic therapy. It is aimed at evaluating whether or not PCI improves survival by safely decreasing the incidence of CNS metastases. The study was recently closed to accrual and the patients will be followed for two years in concert with the primary end-point.

Several factors have been associated with an increased risk of BM in both stage IIIA and IIIB NSCLC after combination therapies, including histology (epidermoid vs. non-epidermoid), gender (female vs. male), age at presentation (younger vs. older than 50 years), nodal status (pN0 vs. residual nodal disease), performance score (KPS ≥ 70 vs. < 70), duration of survival (long vs. short), and chemotherapy protocol (taxane-platinum combination vs. other platinum-based combinations). [2,15-17,21,24]. In our cohort, it was not possible to test the predictive role of chemotherapy, large cell and adenosquamous histologies, and performance score due to the design of the treatment protocol, which was limited to patients classified by RPA groups. However, among the factors that could be analyzed, only age younger than 60 years was associated with higher risk of BM incidence following PCI. Additionally, there was a trend towards a higher risk of BM in patients with N2-3 stage compared to N0-1, but this difference did not reach statistical significance (p = 0.07), which may be due to either the small size of the study population and/or the short duration of the median follow-up interval. Despite of the inherent disadvantages, these findings appear to strongly well correlate with prior reports.

Grouping of patients according to RPA is an established method for prediction of survival in patients with LA-NSCLC. The role of RPA classification in predicting BM development in patients with NSCLC was studied by Komaki et al. [19]. by reviewing the data from four previously reported RTOG studies. Analysis showed that patients included in RPA Groups 1 and 2 with the longest survival had the highest incidence of brain relapses (18% vs. 9%, p = 0.0004). To our knowledge, none of the previously reported PCI trials specifically studied a certain RPA group, and may have included patients belonging to different RPA groups. This raises the suspicion whether the BMFS might be underestimated by short OS due to deaths caused by extracranial metastases. Although our median follow-up was relatively short, the current finding of almost equal BMFS and OS (16.6 vs. 16.7, respectively) in RPA Group 1 patients lends further support to the expectation of a longer survival for patients with LA-NSCLC with reduced risk of BM. We consider that our median survival data of 16.7 month is similar to RTOG studies which resulted of 16.2 months for RPA group 1 patients, [20]. and is at least similar to most of the recent chemoradiotherapy studies published for LA-NSCLC.[25].

One important limitation of our current study is the absence of the comparative neurocognitive assessments in pre- and post-PCI periods. In previous retrospective studies, controversial findings have been reported. [27-31]. In the meta-analysis of PCI in SCLC, [26]. two trials have evaluated the effect of PCI on neurocognitive functions. [27,28]. Arriagada et al.[32]. reported no significant differences in neurocognitive function between patients receiving PCI (24 Gy in 8 fractions) and those in the observation group. Gregor et al. [31]. randomized patients to PCI or no PCI. They reported that there was an impairment of cognitive function and QOL before PCI, and further impairment at 6 and 12 months in both groups. No additional impairment was associated with PCI. None of other randomized trials using PCI, except for the recent German trial, [14]. collected detailed prospective data on the long-term effects of PCI on neurocognitive function in patients with NSCLC. An important limitation of the German trial was the absence of baseline neurocognitive testing. Nevertheless, no significant differences were detected in the patient cohort receiving PCI in comparison with those without PCI despite a detailed neurocognitive investigation in long-term survivors, more than 4 years after treatment. Based on the current evidence from SCLC and NSCLC studies, it is reasonable to assume that PCI regimens consisting of 20–30 Gy given in 2–3 Gy per fractions, as used in the current cohort, will produce little toxicity on brain tissue for the first 1 to 2 years after PCI. However, further studies with longer follow-up and sequential pre- and post-PCI evaluations are needed to fully assess late toxicity of PCI on brain tissue. One hopes that the recent RTOG 0214 protocol will resolve many unanswered issues, including the possible changes in neurocognitive function following PCI.

## Conclusion

PCI as administered in our protocol demonstrated efficacy in reducing the overall incidence of BM and the rate at which the brain is the first site of relapse; our findings also suggest a longer survival for selected patients with LA-NSCLC. We believe that it is necessary to research the role of PCI in RPA group 1 patients with LA-NSCLC in randomized clinical trials with larger patient populations; such studies may provide further data that may help select patients who are most likely to benefit from PCI.

## Competing interests

The authors declare that they have no competing interests.

## Authors' contributions

AAY conceived of the study and wrote the manuscript. ET collected the samples and patient's clinical data. CO and MNY participated in the design of the study and helped write the paper.

All authors read and approved the final manuscript.
